# Efficacy and Safety of Tedizolid Phosphate versus Linezolid in a Randomized Phase 3 Trial in Patients with Acute Bacterial Skin and Skin Structure Infection

**DOI:** 10.1128/AAC.02252-18

**Published:** 2019-06-24

**Authors:** Xiaoju Lv, Jeff Alder, Li Li, William O’Riordan, Michael J. Rybak, Hui Ye, Ruiping Zhang, Zhongqi Zhang, Xu Zhu, Mark H. Wilcox

**Affiliations:** a Centre of Infectious Diseases, West China Hospital, Sichuan University, Chengdu, China; b Bayer HealthCare Pharmaceuticals, Whippany, New Jersey, USA; c Bayer AG, Berlin, Germany; d eStudy Site, Inc., San Diego, California, USA; e Eugene Applebaum College of Pharmacy and Health Sciences, Wayne State University School of Medicine, Detroit, Michigan, USA; f Bayer HealthCare, Beijing, China; g Leeds Teaching Hospitals and University of Leeds, Leeds, United Kingdom

**Keywords:** Asian patients, Chinese patients, acute bacterial skin and skin structure infection, linezolid, methicillin-resistant *Staphylococcus aureus*, oxazolidinone, randomized controlled phase 3 trial, tedizolid phosphate

## Abstract

Tedizolid phosphate is approved for the treatment of acute bacterial skin and skin structure infection (ABSSSI) caused by Gram-positive bacteria in the United States, Europe, and other countries. In this multicenter, double-blind, phase 3 study, 598 adult ABSSSI patients in China, Taiwan, the Philippines, and the United States were randomized to receive 200 mg of tedizolid, intravenously (i.v.)/orally (p.o.), once daily for 6 days or 600 mg of linezolid, i.v./p.o. twice daily for 10 days. The primary endpoint was early clinical response rate at 48 to 72 h. Secondary endpoints included programmatic and investigator-assessed outcomes at end-of-therapy (EOT) and posttherapy evaluation (PTE) visits. Safety was also evaluated. In the intent-to-treat (ITT) population, 75.3% of tedizolid-treated patients and 79.9% of linezolid-treated patients were early responders (treatment difference, –4.6%; 95% confidence interval [CI], –11.2, 2.2). After exclusion of patients who never received the study drug (tedizolid, *n* = 8; linezolid, *n* = 1; modified ITT), comparable early response rates were observed (tedizolid, 77.4%; linezolid, 80.1%; treatment difference, –2.7%; 95% CI, –9.4, 3.9). Secondary endpoints showed high and similar clinical success rates in the ITT and clinically evaluable (CE) populations at EOT and PTE visits (e.g., CE-PTE for tedizolid, 90.4%; for linezolid, 93.5%). Both drugs were well tolerated, and no death occurred. Eight patients experienced phlebitis with tedizolid while none did with linezolid; hence, drug-related treatment-emergent adverse events were reported in a slightly higher proportion in the tedizolid (20.9%) arm than in the linezolid arm (15.8%). The study demonstrated that tedizolid in a primarily Asian population was an efficacious and well-tolerated treatment option for ABSSSI patients. (This study has been registered at ClinicalTrials.gov under registration no. NCT02066402.)

## Abstract (precis)

Tedizolid phosphate is approved for the treatment of acute bacterial skin and skin structure infection (ABSSSI) caused by Gram-positive bacteria in the United States, Europe, and other countries. In this multicenter, double-blind, phase 3 study, 598 adult ABSSSI patients in China, Taiwan, the Philippines, and the United States were randomized to receive 200 mg of tedizolid, intravenously (i.v.)/orally (p.o.), once daily for 6 days or 600 mg of linezolid, i.v./p.o.

## INTRODUCTION

Methicillin-resistant Staphylococcus aureus (MRSA) remains a challenging pathogen globally, with attributable increased risk of morbidity and/or mortality among patients with severe infections, such as nosocomial pneumonia, surgical site infections, bacteremia, or endocarditis (1–6). MRSA is also a concern in skin and soft tissue infections and in acute bacterial skin and skin structure infections (ABSSSIs) in the United States, Europe, and Asian countries (7–11). The prevalence of MRSA in some areas is considerable (e.g., ≥50%), such as in the United States, Russia, Latin American, and Asian countries and regions (e.g., Japan, Singapore, and Taiwan) (12–14). In China, the reported prevalence of MRSA has shown a declining trend over a 10-year period; however, it remains significant at >40% (15).

Owing to diverse clinical presentations and varying level of severity of acute bacterial skin infections, the U.S. Food and Drug Administration (FDA) recently specified the definition of ABSSSI in order to strengthen the clinical development of antibiotics (8, 16). ABSSSIs include cellulitis, erysipelas, wound infection, and major cutaneous abscess with a lesion size of at least 75 cm^2^ [16], and they are among the most frequent skin infections requiring hospitalization globally (8–10, 17–19). These infections are predominantly caused by Gram-positive bacteria, including streptococci and staphylococci (including MRSA), and occasionally by enterococci or Gram-negative species (7, 8, 10, 11, 20). Considering the risk of MRSA is a key step in the management of ABSSSI patients with respect to selection of the most appropriate antibiotic (20–22), particularly in areas where prevalence is significantly high or relevant risk factors are present (e.g., previous MRSA infection or colonization, previous hospitalization, previous antibiotic use, invasive procedures, or chronic open wounds [20]).

New antibiotics with favorable safety profiles and oral formulations to maximize their benefit for outpatient management (23) are still needed in China for the treatment of ABSSSI and other Gram-positive infections. Tedizolid phosphate (here referred to as tedizolid) administered at 200 mg intravenously (i.v.) and/or orally (p.o.) once daily for 6 days was approved by the FDA and European Medicines Agency (EMA) for the treatment of ABSSSI based on the results of two randomized, double-blind, active-controlled, multicenter, international phase 3 clinical studies (24, 25). These studies demonstrated the noninferiority of tedizolid to linezolid in the early clinical response rate and in all secondary endpoints. In addition, a favorable safety profile in terms of gastrointestinal and hematological side effects was observed (24–28).

The objective of the current study (ClinicalTrials.gov registration no. NCT02066402) was to compare the efficacy and safety of treatment with tedizolid at 200 mg, i.v./p.o., once daily for 6 days to that with linezolid at 600 mg, i.v./p.o., twice daily for 10 days in patients with ABSSSI who were enrolled primarily in Asian countries (China, Taiwan, and the Philippines) and the United States.

(The data in the manuscript were presented in part at the 30th International Congress of Chemotherapy and Infection, Taipei, Taiwan, 24 to 27 November 2017 [29].)

## RESULTS

### Patient disposition.

**FIG 1:**
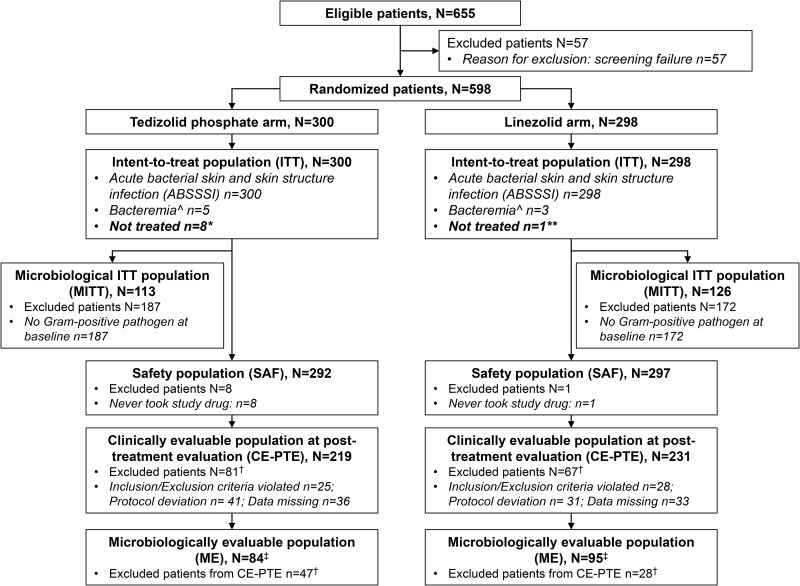
Patient flow through the study. *, withdrew consent (*n* = 7) or noncompliance with study drug (*n* = 1); **, lost to follow-up (*n* = 1); †, patients could have been excluded for more than one reason; ‡, included all patients who were eligible for inclusion in the CE-PTE analysis set with at least one Gram-positive pathogen at baseline; ^, blood culture was negative at screening but positive at a later time point, and the collection of a blood sample was clinically indicated for culture.

Patient flow through the study is shown in Fig. 1. A total of 598 patients with ABSSSI were randomized to receive either tedizolid (*N*= 300) or linezolid (*N* = 298) (intent-to-treat [ITT] population), after exclusion of 57 patients who failed screening (number of patients not meeting all inclusion criteria, 24; number having at least one exclusion criterion, 24; number of withdrawals, 7; number with technical problems, 2) (see Table S1 in the supplemental material).

### Baseline demographics and disease characteristics.

Baseline demographic and infection characteristics were similar between treatment arms (Table 1). The majority of patients (tedizolid arm, 63.7%; linezolid arm, 64.8%) were Asian/Chinese, and approximately one-third of patients were Caucasian. In the ITT population, the majority of patients had cellulitis/erysipelas (Table 1), and most wound infections were posttraumatic wounds (tedizolid arm, *n* = 67; linezolid arm, *n* = 68).

**TABLE 1:** Demographic and baseline characteristics of patients with acute bacterial skin and skin structure infections (intent-to-treat population)

Parameter^*a*^	Value for the treatment group^*b*^	
	Tedizolid phosphate (*N* = 300)	Linezolid (*N* = 298)
Male patients	209 (69.7)	192 (64.4)
Age		
Mean (yr [range])	45.7 (18–85)	47.5 (18–85)
65–75 yr	33 (11.0)	35 (11.7)
>75 yr	8 (2.7)	18 (6.0)
Race		
White	101 (33.7)	93 (31.2)
Asian	191 (63.7)	193 (64.8)
Not reported	2 (0.7)	1 (0.3)
Other	6 (2.0)	11 (3.7)
		
Mean BMI (kg/m^2^ [range])	26.29 (16.3–63.4)^*c*^	25.58 (15.2–50.0)
Comorbidities		
History of diabetes mellitus	26 (8.7)	35 (11.7)
Renal impairment (mild/moderate)	71 (24.1)^*d*^	87 (29.5)^*d*^
Hepatobiliary disorders	19 (6.3)	8 (2.7)
Hepatitis C^*e*^	58 (19.3)	57 (19.1)
Hepatitis B^*f*^	5 (1.7)	9 (3.0)
HIV positive	1 (0.3)	0 (0)
Tinea pedis^*g*^	23 (7.7)	20 (6.7)
Present or recent i.v. drug use^*e*^	89 (29.7)	80 (26.8)
Previous ABSSSI lesion	91 (30.3)	74 (24.8)
Type of primary infection	300 (100)	298 (100)
Primary diagnosis as ABSSSI	300 (100)	298 (100)
Cellulitis or erysipelas	192 (64.0)	191 (64.1)
Wound infections	68 (22.7)	68 (22.8)
Major cutaneous abscess	40 (13.3)	39 (13.1)
ABSSSI with secondary bacteremia	5 (1.7)	3 (1.0)
At least one Gram-positive organism identified at baseline^*h*^	113 (37.7%)	126 (42.3%)
Gram-positive aerobes	113/113 (100)	123/126 (97.6)
Staphylococcus aureus	79/113 (69.9)^*i*^	95/126 (75.4)^*j*^
MSSA	51/113 (45.1)	64/126 (50.8)
MRSA	29/113 (25.7)	32/126 (25.4)
Streptococcus anginosus	23/113 (20.4)	23/126 (18.3)
Streptococcus pyogenes	4/113 (3.5)	6/126 (4.8)
Streptococcus mitis group	4/113 (3.5)	4/126 (3.2)
Polymicrobial Gram-positive infection	13 (11.5)	25 (19.8)
Mixed Gram-positive and Gram-negative infection	5 (4.4)	7 (5.6)
Disease characteristics		
Fever^*k*^	67/292 (22.9)	84/296 (28.4)
WBC count of ≥10,000 or <4,000 cells/mm^3^	153/298 (51.3)	149/297 (50.2)
Immature neutrophils (>10%)	7/160 (4.4)	7/156 (4.5)
Lymphadenopathy	219/299 (73.2)	213/298 (71.5)
Lesion size (cm^2^)		
Median	302.5	306.75
Mean (SD)	491.6 (618.1)	428.3 (391.7)
Range	75.0–6272.0	77.0–2664.0
Anatomical location of ABSSSI^*l*^		
Lower leg	120 (40.0)	115 (38.6)
Foot dorsal	33 (11.0)	32 (10.7)
Thigh	27 (9.0)	25 (8.4)
Buttock	16 (5.3)	16 (5.4)
Head	15 (5.0)	16 (5.4)
Forearm	15 (5.0)	20 (6.7)
Upper arm	16 (5.3)	22 (7.4)
Other^*m*^	60 (20.0)	64 (21.5)
Duration of intravenous treatment (days)		
Median	4.0	3.0
Mean (SD)	4.2 (2.3)	4.0 (2.3)

a ABSSSI, acute bacterial skin and skin structure infection; BMI, body mass index; MRSA, methicillin-resistant Staphylococcus aureus; MSSA, methicillin-susceptible S. aureus; i.v. intravenous; SD, standard deviation; WBC, white blood cell.

b Unless otherwise noted, values represent the number of patients positive for the parameter and the percentage of the total treatment group.

c *N*_1_ = 299.

d *N*_1_ = 295.

e All but one non-Asian patient.

f Five Asian patients in each arm.

g All Asian patients.

h Patients could have had mixed or polymicrobial infection.

i One patient in the tedizolid arm had been infected with both MSSA and MRSA; data are shown by patient.

j One patient in the linezolid arm had been infected with both MSSA and MRSA; data are shown by patient.

k Body temperature 38°C (oral), 38.5°C (tympanic), or 39°C (rectal).

l Patients could have had multiple anatomical sites provided they were contiguous.

m Includes (but is not limited to) the following: face, hand dorsal, shoulder, abdomen, axilla, back, limb, etc.

The percentages of patients with a confirmed pathogen at baseline (microbiological ITT [MITT] population) were similar between treatment arms (tedizolid, 37.7%; linezolid, 42.3%), and of those, 25.7% in the tedizolid arm and 25.4% in the linezolid arm had MRSA at baseline (Table 1). The rate of isolation of Gram-positive pathogens was the lowest among patients with cellulitis (tedizolid arm, 16.7%; linezolid arm, 18.3%) and much higher among patients with major cutaneous abscess (tedizolid arm, 62.5%; linezolid arm, 79.5%) or with wound infections (tedizolid arm, 82.4%; linezolid arm, 88.2%).

Although the median lesion sizes were similar between treatment arms, the mean and range of lesion sizes were greater in the tedizolid arm than in the linezolid arm (Table 1); however, the difference was not significant. The difference in mean values of lesion size at baseline was more prominent in patients with confirmed pathogens than in those with suspected pathogens (Table S2). A low proportion of patients received aztreonam (tedizolid arm, 3.3%; linezolid arm, 5.7%) or metronidazole (tedizolid arm, 1.0%; linezolid arm, 1.0%), which were permitted concomitant antibiotics according to the protocol.

### Primary endpoint.

In the ITT population, 226 patients out of 300 (75.3%) in the tedizolid arm and 238 patients out of 298 (79.9%) in the linezolid arm achieved the early clinical response (i.e., ≥20% reduction in lesion size, no concomitant antibiotic used, and no death occurred) at 48 to 72 h. The difference in early clinical response rates between treatment arms was –4.6%, with the lower boundary of the 95% confidence interval (CI) being below –10% (i.e., 95% CI, –11.2%, 2.2%), indicating that the noninferiority criterion was not met (Table 2). Eight patients in the tedizolid arm due to consent withdrawal (*n* = 7) or noncompliance with study drug (*n* = 1) and one patient in the linezolid arm due to loss to follow-up did not receive the study drug after randomization and were therefore considered as having an indeterminate response (ITT population).

**TABLE 2:** Primary and secondary efficacy endpoints in different analysis populations

Parameter^*a*^	Tedizolid phosphate^*b*^		Linezolid^*c*^		Treatment difference (95% CI)
	Group size	Value for the parameter (no. [%])^*d*^	Group size	Value for the parameter (no. [%])^*d*^	
Programmatic early clinical response at 48–72 h in ITT population (*n*/*N* [%])	*N* = 300		*N* = 298		
Responder		226 (75.3)		238 (79.9)	–4.6% (−11.2%; 2.2%)
Nonresponder or indeterminate		74 (24.7)		60 (20.1)	
Nonresponder		51 (17.0)		41 (13.8)	
Indeterminate		23 (7.7)		19 (6.4)	
Programmatic early clinical response at 48–72 hours in mITT population (*n*/*N* [%])	*N* = 292		*N* = 297		
Responder		226 (77.4)		238 (80.1)	–2.7% (–9.4%; 3.9%)
Nonresponder or indeterminate		66 (22.6)		59 (19.9)	
Nonresponder		51 (17.5)		41 (13.8)	
Indeterminate		15 (5.1)		18 (6.1)	
Programmatic clinical response at EOT in ITT population (*n*/*N* [%])	*N* = 300		*N* = 298		
Clinical success		246 (82.0)		251 (84.2)	–2.2% (–8.3%; 3.8%)
Clinical failure or indeterminate		54 (18.0)		47 (15.8)	
Clinical failure		34 (11.3)		30 (10.1)	
Indeterminate		20 (6.7)		17 (5.7)	
Programmatic clinical response at EOT in CE-EOT population (*n*/*N* [%])	*N* = 242		*N* = 243		
Clinical success		217 (89.7)		223 (91.8)	–2.1% (–7.4%; 3.2%)
Clinical failure		25 (10.3)		20 (8.2)	
Investigator’s assessment of clinical response at PTE visit in ITT population (*n*/*N* [%])	*N* = 300		*N* = 298		
Clinical success		239 (79.7)		244 (81.9)	–2.2% (–8.6; 4.1)
Clinical failure or indeterminate		61 (20.3)		54 (18.1)	
Clinical failure		25 (8.3)		21 (7.0)	
Indeterminate		36 (12.0)		33 (11.1)	
Investigator's assessment of clinical response at PTE in CE-PTE population (*n*/*N* [%])	*N* = 219		*N* = 231		
Clinical success		198 (90.4)		216 (93.5)	–3.1% (–8.4; 2.0)
Clinical failure		21 (9.6)		15 (6.5)	

a CE, clinically evaluable; CI, confidence interval; EOT, end of therapy; ITT, intent to treat; mITT, modified ITT (i.e., exclusion of patients who never received study drug); PTE, posttherapy evaluation.

b Dosed at 200 mg once daily for 6 days.

c Dosed at 600 mg twice daily for 10 days.

d Values represent number of patients (%) in the group.

In the *post hoc* analysis of the modified ITT population (mITT; after exclusion of eight randomized patients in the tedizolid arm and one randomized patient in the linezolid arm who did not receive any study drug), comparable early clinical response rates were demonstrated between treatment arms at 48 to 72 h (tedizolid arm, 77.4%; linezolid arm, 80.1%; treatment difference, –2.7%; 95% CI, –9.4%, 3.9%) (Table 2).

### Secondary endpoints.

Analyses of the programmatic and investigator assessment of clinical success rates demonstrated that all prespecified secondary endpoints were met (Table 2). Comparable efficacies between tedizolid and linezolid were demonstrated at end of therapy (EOT) in the ITT population (tedizolid arm, 82.0%; linezolid arm, 84.2%; treatment difference, –2.2%; 95% CI, –8.3%, 3.8%) and in the clinically evaluable (CE)-EOT population (tedizolid arm, 89.7%; linezolid arm, 91.8%; treatment difference, –2.1%; 95% CI, –7.4%, 3.2%) for the programmatic clinical outcome. Furthermore, rates of investigator assessment of clinical success were comparable at the posttherapy evaluation (PTE) visit in the ITT (tedizolid arm, 79.7%; linezolid arm, 81.9%; treatment difference, –2.2%; 95% CI, –8.6%; 4.1%) and CE-PTE populations (tedizolid arm, 90.4%; linezolid arm, 93.5%; treatment difference, –3.1%; 95% CI, –8.4%, 2.0%).

In both treatment arms, improvement in the overall clinical status that was compatible with continuation of therapy was seen in a similar proportion of patients at 48 to 72 h (tedizolid arm, 89.0%; linezolid arm, 90.6%) and at day 7 (tedizolid arm, 90.8%; linezolid arm, 87.9%) based on investigator assessment in the modified ITT population (Table S3) and also in the ITT population (data not shown).

### Investigator assessment of clinical signs and symptoms.

Investigator assessment of systemic, regional, and local signs and symptoms of ABSSSI were evaluated in *post hoc* analyses in the mITT population. The numbers of patients with a valid assessment at baseline for all investigated parameters were comparable between treatment arms. The results demonstrated that over the course of the study period, the degrees of improvements in all signs and symptoms (i.e., severity of lymph node tenderness, lymphadenopathy, erythema, edema, and induration) were similar in the tedizolid and linezolid arms (Table S4). Tedizolid and linezolid treatments resulted in similar absolute reductions in the mean lesion size at sequential time points (day 2, 48 to 72 h; day 7, and EOT and PTE visits) although slightly greater reductions were observed in tedizolid-treated patients than in linezolid-treated patients with confirmed pathogen at baseline in *post hoc* analyses (Table S5).

At baseline, a small proportion of patients in both treatment arms (tedizolid arm, 22.9%; linezolid arm, 28.4%) had fever. The median times to resolution of fever were 41.2 h in the tedizolid arm and 40.9 h in the linezolid arm. Changes in patient-reported pain scores from baseline were similar between the two arms, as measured by either visual analogue scale (VAS) or faces rating scale (FRS) scoring (Table S6).

### Efficacy results by infection type.

In the ITT population at 48 to 72 h, the early clinical response rate was numerically higher in the tedizolid arm than in the linezolid arm (tedizolid arm, 95.0%; linezolid arm, 84.6%) among patients with major abscess. The early clinical response rate was numerically lower in the tedizolid arm than in the linezolid arm among patients with cellulitis/erysipelas (tedizolid arm, 70.3%; linezolid arm, 78.0%) and wound infection (tedizolid arm, 77.9%; linezolid arm, 82.4%). In the CE-PTE population at the PTE visit, investigator assessment of clinical success was demonstrated with 6-day tedizolid and 10-day linezolid treatments, respectively, in 97.0% and 96.4% of patients with major abscess, in 86.9% and 92.1% of patients with cellulitis/erysipelas, and in 95.9% and 96.1% of patients with wound infection.

### Microbiological results.

**FIG 2:**
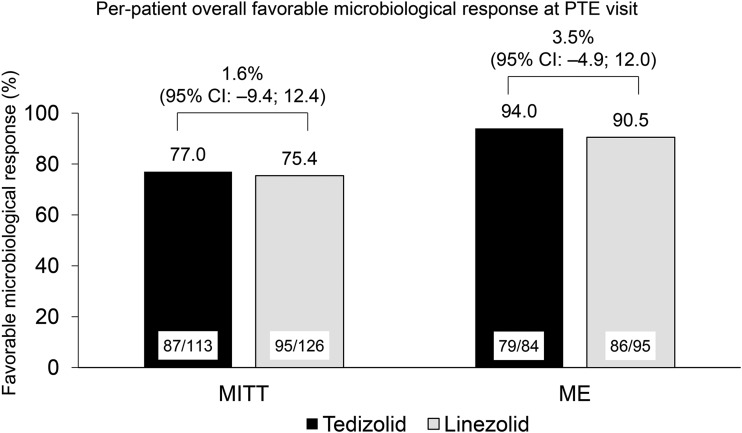
Per-patient overall favorable microbiological response at posttherapy evaluation visit (PTE). CI, confidence interval; ME, microbiological evaluable; MITT, microbiological intent to treat. A favorable response was equivalent to “presumed eradication” for the different baseline infection types.

All S. aureus, methicillin-susceptible S. aureus (MSSA), MRSA, and other Gram-positive pathogens had tedizolid MICs of ≤0.5 μg/ml and linezolid MICs of ≤4 μg/ml. For both MRSA and MSSA, the tedizolid MIC ranged between 0.25 and 0.5 μg/ml in the tedizolid arm, and the linezolid MIC ranged between 1.0 and 4.0 μg/ml in the linezolid arm. Overall, the rates of favorable microbiological responses were similar in the two treatment arms in the MITT (tedizolid arm, 77.0%; linezolid arm, 75.4%; treatment difference, 1.6%; 95% CI, –9.4%, 12.4%) and microbiologically evaluable (tedizolid arm, 94.0%; linezolid arm, 90.5%; treatment difference, 3.5%; 95% CI, –4.9%, 12.0%) populations for patients with a confirmed Gram-positive pathogen at baseline (Fig. 2). At the PTE visit, the rates of investigator assessment of clinical success by pathogen were high although some numerical differences between treatment arms were seen due to low patient numbers (Table 3). In an exploratory *post hoc* analysis, evaluation of early clinical response for patients with confirmed pathogen at baseline in the MITT and modified MITT populations showed similar rates between the tedizolid and linezolid treatment arms (78.8% for the tedizolid arm [*n* = 113] and 81.0% for the linezolid arm [*n* = 126] in the MITT population; 80.9% for the tedizolid arm [*n* = 110] and 81.6% for the linezolid arm [*n* = 125] in the modified MITT population). These *post hoc* analyses data should be interpreted with caution.

**TABLE 3:** Investigator assessment of clinical success at the posttherapy evaluation visit by baseline pathogen in the microbiological intent-to-treat population

Species^*a*^	No. of patients with clinical success/no. of patients with pathogen confirmed at baseline (%)	
	Tedizolid phosphate (*N* = 113)	Linezolid (*N* = 126)
Staphylococcus aureus^*b*^	60/79 (75.9)	70/95 (73.7)
MSSA	40/51 (78.4)	51/64 (79.7)
MRSA	21/29 (72.4)	20/32 (62.5)
Streptococcus anginosus	21/23 (91.3)	18/23 (78.3)
Streptococcus pyogenes	2/4 (50.0)	5/6 (83.3)
Streptococcus mitis group	4/4 (100)	4/4 (100)

a Other Gram-positive pathogens in very small numbers included the following: Staphylococcus haemolyticus, Streptococcus canis, Staphylococcus lugdunensis, Streptococcus agalactiae, Enterococcus faecalis, *Enterococcus* spp., Gemella morbillorum, Mycobacterium fortuitum, Clostridium tertium, Finegoldia magna, and Propionibacterium avidum. MRSA, methicillin-resistant Staphylococcus aureus; MSSA, methicillin-susceptible S. aureus.

b One patient in the tedizolid arm and one patient in the linezolid arm had been infected with both MSSA and MRSA; data are shown by patient.

### Safety findings.

Both tedizolid and linezolid treatments were well tolerated. The incidence of treatment-emergent adverse events (TEAEs) was comparable between the tedizolid (49.7%) and linezolid (45.8%) arms, and most TEAEs were mild or moderate in intensity (Table 4). Numerically, a higher proportion of tedizolid-treated patients had drug-related TEAEs than linezolid-treated patients (20.9% versus 15.8%, respectively) (Table 4). The proportions of patients experiencing serious TEAEs and treatment discontinuation were low and similar between treatment arms. No drug-related serious TEAE or death occurred in either treatment arm. Discontinuation due to serious TEAEs occurred only in the linezolid arm (*n* = 3) (Table 4).

**TABLE 4:** Overall safety: treatment-emergent adverse events (safety population)

TEAE type^*a*^	Value for the group (no. of patients [%])	
	Tedizolid phosphate (*N* = 292)^*b*^	Linezolid (*N* = 297)^*c*^
Any event	145 (49.7)	136 (45.8)
Mild	95 (32.5)	89 (30.0)
Moderate	37 (12.7)	40 (13.5)
Severe	12 (4.1)	7 (2.4)
Missing	1 (0.3)	0 (0)
Drug-related event	61 (20.9)	47 (15.8)
Serious event	11 (3.8)	8 (2.7)
Drug-related serious event	0 (0)	0 (0)
Event leading to discontinuation of study drug	6 (2.1)	6 (2.0)
Serious event leading to discontinuation of study drug	0 (0)	3 (1.0)
Any event leading to death	0 (0)	0 (0)

a TEAE, treatment-emergent adverse event.

b Dosed 200 mg once daily for 6 days.

c Dosed at 600 mg twice daily for 10 days.

The summary of drug-related adverse events occurring in ≥1% of patients in either treatment arm is shown in Table 5. Of note, in the tedizolid arm, eight patients (2.7%) experienced phlebitis that was related to the study drug whereas none did in the linezolid arm. Other drug-related TEAEs occurred at similar rates between the tedizolid and linezolid arms (Table 5). The rates of overall gastrointestinal (GI) TEAEs were low and comparable in the two treatment arms (tedizolid arm, 8.9%; linezolid arm, 10.4%).

**TABLE 5:** Drug-related treatment-emergent adverse events occurring in ≥1% of patients in either treatment arm (safety population)

System organ class and preferred term^*a*^	Value for the group (no. of patients [%])	
	Tedizolid phosphate (*N* = 292)^*b*^	Linezolid (*N* = 297)^*c*^
Gastrointestinal disorders		
Nausea	11 (3.8)	11 (3.7)
Diarrhea	4 (1.4)	3 (1.0)
Vomiting	4 (1.4)	2 (0.7)
General disorders and administration site conditions		
Fatigue	1 (0.3)	3 (1.0)
Nervous system disorders		
Headache	2 (0.7)	4 (1.3)
Hepatobiliary disorders		
Hepatic function abnormal	2 (0.7)	4 (1.3)
Vascular disorders		
Phlebitis reported	8 (2.7)	0

a Any one patient is counted only once within each preferred term of any primary system organ class.

b Dosed at 200 mg once daily for 6 days.

c Dosed at 600 mg twice daily for 10 days.

Rates of substantially abnormal findings in laboratory investigations are shown in Table S7. Levels of the hepatic enzymes alanine aminotransferase (ALT) and aspartate aminotransferase (AST) were elevated in a numerically higher proportion of patients treated with linezolid (ALT, 7.2%; AST, 4.6%) than with tedizolid (ALT, 5.1%; AST, 2.2%) (Table S7). Hematological findings in the safety population showed that a very low number of patients in both treatment arms experienced substantially abnormal values of hemoglobin (tedizolid arm, 0 patients; linezolid arm, 2), absolute neutrophil count (tedizolid arm, 0; linezolid arm, 3), or platelet count (tedizolid arm, 2; linezolid arm, 1) (Table S7). The proportions of patients with abnormal platelet values (including those with normal or abnormal baseline values and nonmissing data at the subsequent visits) were also low and comparable in the two treatment arms at days 7 to 9 (2.1% [5/234] for tedizolid versus 0% [0/218] for linezolid), at days 11 to 13 (4.4% [11/250] for tedizolid versus 3.4% [8/236] for linezolid), at the last dose of active drug (2.1% [5/234] for tedizolid versus 3.4% [8/236] for linezolid), and at any postbaseline visit through to last dose of active drug (3.4% [8/234] for tedizolid versus 4.5% [10/224] for linezolid).

There were no remarkable findings for vital signs, electrocardiogram findings, Clostridium difficile-associated diarrhea, lactic acidosis, and neurological assessments.

## DISCUSSION

This was the third international, multicenter, randomized, double-blind, double-dummy phase 3 controlled study comparing the efficacy and safety of tedizolid at 200 mg once daily for 6 days and linezolid at 600 mg twice daily for 10 days in patients with ABSSSI, who were primarily enrolled in Asian countries. The ESTABLISH-1 (p.o. only) and -2 (i.v./p.o., switching from i.v. to p.o. when meeting criteria) studies previously demonstrated the noninferiority of tedizolid to linezolid in terms of the early clinical response rate at 48 to 72 h and in clinical success rates at later time points (24–26).

The findings of the current study demonstrated that the noninferiority of tedizolid to linezolid in the early clinical response rate (i.e., ≥20% reduction in lesion size from baseline within 72 h after first infusion of study drug was required in the ITT population) was inconclusive. The lower limit of the 95% CI was below –10% in the ITT population (including all randomized patients), indicating that noninferiority was not met. However, an imbalance in the number of randomized but untreated patients was observed (8 for tedizolid versus 1 for linezolid) in this study. After excluding in a *post hoc* analysis these patients who never received a study drug, comparable early clinical response rates at 48 to 72 h were demonstrated between tedizolid and linezolid treatment arms in the modified ITT population. At later protocol-specified time points, the rates of sustained programmatic clinical response at EOT and investigator assessment of clinical success at the PTE visit (which is the primary endpoint defined by EMA) were comparable between the two treatment arms. Both treatments were well tolerated, and the study did not reveal any new safety signals with tedizolid treatment in this primarily Asian population, with the exception of a higher reported rate of drug-related phlebitis in the tedizolid arm.

The design of the current study, closely resembling that of ESTABLISH-2 (25), allowed the switch from i.v. to p.o. therapy at the discretion of the treating physician. A phase 1 study conducted in healthy Chinese individuals demonstrated a high oral bioavailability of tedizolid (i.e., 85.5%), suggesting that no dose adjustment is needed when therapy is switched from i.v. to p.o. in Chinese patients (30). The mean duration of i.v. treatment was approximately 4.2 days, and compliance to study drug was high in both treatment arms.

The patient population enrolled in the current study had some numerical differences in baseline demographic parameters and disease characteristics compared with those of patients enrolled in the ESTABLISH studies. Thus, the proportion of patients with cellulitis/erysipelas was 64% in the current study whereas it was 45% in ESTABLISH-1 and -2 [24–26].

Additionally, there was a nonsignificant difference between the two treatment arms in the ranges of lesion sizes at baseline (tedizolid arm, 75 to 6,272 cm^2^; linezolid arm, 77 to 2,664 cm^2^). There is no defined maximum lesion size for enrollment into ABSSSI studies (16). Furthermore, this was the first study in China using ≥20% reduction in lesion size, measured by the ruler method, at 48 to 72 h as the primary endpoint, which may limit comparison with other trials. Despite the imbalance in the mean and maximum lesion sizes, the investigators demonstrated in the mITT population a similar trend between the two treatment arms in the absolute reduction of lesion size from day 2 up to the PTE visit, which may indicate continuing improvements in local signs and symptoms even in patients with very large lesions (e.g., cellulitis/erysipelas with an area of 6,272 cm^2^). The lesion size reductions were complemented by similar trends in improvements of lymphadenopathy, time to resolution of fever, changes in pain scores, and declining severity of erythema, edema, and induration among randomized and treated patients, suggesting that patients responded to both treatments from an early time point.

In real-life clinical practice, a change in antibiotic treatment is recommended if signs and symptoms of skin infections do not improve or if the patient deteriorates at an early stage of management and/or when microbiological information becomes available and escalation or deescalation of antibiotic treatment is required (31). In the current study, the blinded investigators continued the study drug therapy based on the overall assessment of clinical status in approximately 90% of patients in both treatment arms at 48 to 72 h and at day 7. Furthermore, the number of patients who discontinued therapy due to TEAEs was low in both treatment arms. These findings may reflect current clinical practice (32).

The mismatch between the proportion of patients who were early responders according to the protocol and achieved ≥20% reduction in lesion size and those patients with gradual improvements in overall clinical status of ABSSSI from day 2 up to the PTE visit might correspond with the findings by Nathwani et al. (33). Integrated analyses of the ESTABLISH studies suggested that early clinical response was highly predictive of late clinical success at the PTE visit; however, the lack of early clinical response correlated poorly with clinical failure at later time points (32, 33). Thus, the clinical decision to continue therapy when patients improve in their clinical status may supersede the evaluation of a single parameter (i.e., lesion size measurement only). Similar findings were observed in the ESTABLISH-2 study in terms of continuation of therapy by blinded investigators (25).

The high clinical success rates seen in the current study with 6-day tedizolid treatment in the clinically evaluable population at EOT (89.7%) and PTE (90.4%) visits were in agreement with those in the ESTABLISH-1 and -2 studies (80.2% at EOT and 94.6% at PTE in the ESTABLISH-1 study; 90.0% at EOT and 92.0% at PTE in the ESTABLISH-2 study) (24, 25). Analysis of the integrated ESTABLISH studies by Sandison et al. demonstrated that, regardless of the severity of the baseline disease characteristics (e.g., presence or absence of fever, lymphadenopathy, and elevated white blood cell count), both tedizolid and linezolid achieved high clinical success rates at the PTE visit (34). Furthermore, at the PTE visit, high clinical success rates (current study, 86.9% to 97.0%; ESTABLISH-1, 93.2% to 97.4%; ESTABLISH-2, 91.0% to 95.0%) were seen in all three studies in all infection types (i.e., cellulitis/erysipelas, wound infection, or major abscess), suggesting consistent comparable clinical efficacy of 6-day tedizolid in patient populations with high protocol and treatment compliance across geographical regions.

The proportion of any confirmed baseline pathogen (∼40.0%) or MRSA (∼25%) was lower in this study than in the ESTABLISH studies (∼62% and ∼35%, respectively). The high proportion of cellulitis/erysipelas in this study limited the number of patients with a confirmed pathogen at baseline as biospecimens taken from cellulitis patients rarely yield a pathogen (35). Despite a lower yield of confirmed pathogens, rates of eradication and of presumed eradication against most pathogens were comparable between tedizolid and linezolid treatments in both the MITT and ME populations. The high eradication rates were supported by the 100% susceptibility of baseline pathogens to tedizolid and translated into high rates of clinical success assessed by the investigator; these results are similar to those observed in previous ABSSSI studies with tedizolid (24, 25). Global ongoing surveillance studies report that susceptibility to tedizolid of S. aureus, MRSA, beta-hemolytic streptococci, viridans group streptococci, and enterococci, isolated from skin biospecimens or blood, is at least 4-times greater than that to linezolid (36, 37), suggesting that tedizolid could be an effective choice for the treatment of Gram-positive infections.

Both treatments were well tolerated, and no new safety signal was reported in this primarily Asian population. A slightly higher incidence of drug-related TEAEs was observed with tedizolid than with linezolid treatment, which was attributed to the higher number of patients (*n* = 8) experiencing phlebitis at the infusion site (without discontinuation and considered not serious events) than in the linezolid arm (*n* = 0). This rate (20.9%) of drug-related TEAEs in the tedizolid arm of the current study was similar to that of the integrated ESTABLISH studies (22.4%) (27). Furthermore, the overall rates of GI TEAEs (including those related to study drug) and abnormal hematological findings were lower in both treatment arms than in the integrated data from the ESTABLISH studies (26). However, the lower limit of the normal value of the platelet count was different from that used in the previous studies, and therefore results of the incidence of thrombocytopenia must be viewed with caution when comparing ABSSSI studies.

The favorable safety profile of i.v./p.o. tedizolid treatment at 200 mg once daily, for approximately 10 days, has recently been demonstrated in a phase 3 clinical study in Japanese patients with complicated skin and soft tissue infections in terms of hematological and GI drug-related TEAEs versus linezolid (38). Additionally, according to a case series publication, tedizolid treatment for an extended duration of 7 to 14 days in four severe and complex ABSSSI patients was effective and well tolerated without any reported adverse event (39).

### Conclusion.

In conclusion, the study demonstrated that treatment with tedizolid at 200 mg once daily for 6 days achieved a consistent level of clinical efficacy in a primarily Asian/Chinese ABSSSI patient population comparable to treatment with linezolid. Tedizolid treatment was well tolerated without any previously unidentified adverse event, and only a low risk of hematological or GI side effects was observed. Tedizolid at 200 mg once daily for 6 days, with a potential switch from i.v. to p.o. therapy, seems to be an appropriate choice for the treatment of Chinese or other Asian patients diagnosed with ABSSSI.

## MATERIALS AND METHODS

### Study design.

This was a randomized (1:1), double-blind, double-dummy, multicenter, active-controlled, noninferiority phase 3 clinical registration study enrolling patients with ABSSSI. The study was conducted between 4 March 2014 and 18 April 2016 in 39 centers in China, 6 centers in Taiwan, 2 centers in the Philippines, and 5 centers in the United States. This study was registered with ClinicalTrials.gov under registration number NCT02066402.

### Ethical regulations.

All patients or their legal representative provided written consent to participate in the study. The study was conducted according to the Declaration of Helsinki, and approval of the clinical study protocol from local ethical committees or institutional review boards was obtained at all centers according to Good Clinical Practice guidelines, local ethical laws, regulations, and/or organizations.

### Inclusion criteria.

Adult (age of ≥18 years) male or female patients were eligible for enrollment if they were diagnosed with ABSSSI (i.e., cellulitis, erysipelas, major cutaneous abscess, or wound infection [superficial incision surgical site occurring within 30 days following only clean surgery or posttraumatic infection]) caused by suspected or confirmed Gram-positive bacteria, who required i.v. antibiotic therapy, had adequate access for at least two i.v. doses of study drug, and if their local symptoms started within 7 days prior to screening. The minimum lesion size for all three ABSSSI types was 75 cm^2^ (measured head to toe, length by width by flexible ruler method).

All patients had at least one of the following regional or systemic signs of infection: (i) lymph node tenderness and increase in volume or palpable proximal to the primary ABSSSI (lymphadenopathy), (ii) fever, (iii) white blood cell count of ≥10,000 cell/mm^3^ or <4,000 cell/mm^3^ blood, or (iv) >10% immature neutrophils. In patients with cellulitis/erysipelas, at least two of the local signs of infection were present (i.e., erythema, edema, induration, localized warmth, and pain or tenderness on palpation). In patients with major cutaneous abscess, the presence of pus in the dermis or deeper was accompanied within 24 h by erythema, edema, and/or induration extending ≥5 cm in the shortest distance from the peripheral margin of the abscess and also by at least one of the following signs: (i) fluctuance, (ii) incision and drainage required, (iii) purulent or seropurulent drainage, (iv) localized warmth, and (v) pain or tenderness on palpation. In patients with wound infection, the presence of purulent drainage was accompanied by erythema, edema, and/or induration extending ≥5 cm in the shortest distance from the peripheral margin of the wound. A biospecimen taken by aspiration, biopsy, incision, or deep swab was required for patients with major abscess or wound infection.

### Key exclusion criteria.

Key exclusion criteria were uncomplicated skin and skin structure infection (e.g., minor abscess, minor wound infection, and impetiginous lesion); infection associated with a prosthetic device or a vascular catheter or thrombophlebitis; systemic antibiotic therapy with activity against Gram-positive bacteria within 24 h prior to the first infusion of study drug; and confirmed Gram-negative bacteria in association with the ABSSSI, except for patients with wound infection, who were allowed to be treated with concomitant systemic aztreonam and/or metronidazole to cover aerobic and/or anaerobic Gram-negative pathogens. Exclusion criteria are listed in full in the supplemental material.

### Treatments.

Patients were randomized to receive i.v./p.o. tedizolid phosphate at 200 mg once daily for 6 days followed by placebo for 4 days or i.v./p.o. linezolid at 600 mg twice daily for 10 days. A minimum of two doses of study drug was administered as an i.v. infusion, and, at the discretion of the treating investigator, the patient could be switched to p.o. treatment for the rest of the treatment duration.

In order to maintain blinding, a double-dummy treatment design was used: patients in the tedizolid arm received one active dose of tedizolid plus two doses of placebo-linezolid; patients in the linezolid arm received two active doses of linezolid plus one dose of placebo-tedizolid. Doses were administered either as i.v. infusion or p.o. tablet. All patients, the investigators, hospital staff and nurses responsible for patient care and clinical evaluations, and the sponsor were blinded to treatments.

### Patient populations.

The efficacies of tedizolid and linezolid were compared in the intent-to-treat (ITT), clinically evaluable (CE), microbiological ITT (MITT), and microbiologically evaluable (ME) populations. The ITT population comprised all randomized patients assigned to either treatment arm. The CE population included patients who received all study treatments without major protocol violation, did not receive any concomitant potentially effective antibiotic treatment, and completed the assessment at the end-of-therapy visit (CE-EOT) and/or at the posttherapy evaluation visit (CE-PTE). The MITT population comprised all ITT randomized patients with a confirmed Gram-positive pathogen at baseline. The ME population comprised all patients valid for the CE-PTE population who had a confirmed Gram-positive pathogen at baseline. The safety of tedizolid and linezolid was compared in the safety (SAF) population, which included all randomized patients who received at least one dose of study drug in either treatment arm.

The efficacy of tedizolid and linezolid was also compared in a *post hoc* analysis in the modified intent-to-treat population (mITT), which excluded patients who did not receive any study drug.

### Endpoints and definition of clinical outcomes.

The primary objective of the study was to demonstrate that i.v./p.o. tedizolid treatment at 200 mg once daily for 6 days was noninferior to i.v./p.o. linezolid treatment at 600 mg twice daily for 10 days in the early clinical response at 48 to 72 h in the ITT population.

Patients were evaluated as responders or nonresponders to therapy. The early clinical response was defined as ≥20% reduction in lesion size (length by width of erythema, edema, and/or induration from head to toe, measured with a flexible ruler) in a patient who did not receive any prohibited concomitant systemic antibiotic and did not die within 72 h after the first infusion of study drug. Patients were evaluated as nonresponders if any of the following criteria were met: (i) <20% reduction in lesion size compared with baseline; (ii) administration of any systemic concomitant antibiotic with activity against the baseline pathogen within 72 h after the first infusion of study drug; (iii) death occurring within 72 h after the first infusion of study drug. Patients with missing data for the primary objective were considered nonresponders. Patients for whom clinical response could not be determined were considered indeterminate and calculated as nonresponders in the primary outcome analysis.

The secondary planned objectives of the study included programmatic and/or investigator assessment of clinical outcomes (i.e., clinical success, clinical failure, or indeterminate) at the EOT and PTE visits. Full detailed definitions of clinical responses are described in the supplemental material. The rates of sustained (programmatic) objective clinical response in both treatment arms were compared at the EOT visit in the ITT population and the CE-EOT population. The rates of investigator assessment of clinical success at the EOT and PTE visits in the ITT and CE-EOT or CE-PTE populations, respectively, were also compared. Patients in whom treatment outcomes were considered by the investigator to be a clinical failure at the EOT visit were carried forward as clinical failures to the PTE visit in the ITT and the CE-PTE populations.

Prespecified other endpoints included investigator assessment of changes in systemic (i.e., fever, white blood cell count, and immature neutrophils), regional (i.e., lymph node tenderness and lymphadenopathy), and local (i.e., lesion size, erythema, edema, fluctuance, induration, pain to palpation, drainage, and localized warmth) signs and symptoms of infection over the course of the study (i.e., day 1, day 2, 48 to 72 h, day 7, EOT, PTE, and late follow-up). Changes in pain score compared with the baseline level over the course of the study were evaluated by the investigator using a visual analogue scale (VAS) and the Wong-Baker faces rating scale (FRS) methods. Time to resolution of fever was assessed by the investigator.

Other prespecified clinical efficacy endpoints included investigator assessment of clinical success rates overall and per pathogen in both treatment arms in the MITT and ME populations at the PTE visit. Rates of per-patient favorable response and per-pathogen microbiological response in the MITT and ME populations were also assessed at the PTE visit. A favorable microbiological response was defined as eradication and presumed eradication, whereas an unfavorable microbiological response was defined as persistence, presumed persistence, and indeterminate responses.

### Microbiological evaluation.

Baseline pathogens and their antibiotic susceptibility patterns isolated from biospecimens, which were taken by needle aspiration, biopsy, deep swab, or incision from the primary lesion site and/or blood samples, were evaluated in three central laboratories (Covance Central Laboratory Services, Indianapolis, IN, USA; Singapore; and Shanghai, China). Susceptibility of baseline pathogens to tedizolid and linezolid defined by the MIC was determined according to Clinical and Laboratory Standards Institute guidelines (40, 41).

### Safety investigations.

Safety investigations included reporting of adverse events (Medical Dictionary for Regulatory Activities [MedDRA], version 16.0 or higher), laboratory evaluations (e.g., complete blood cell count, hepatic enzymes, renal function, and blood chemistry), vital signs, electrocardiogram parameters, physical examinations, emergence of Clostridium difficile-associated diarrhea, and neurotoxicity evaluations.

### Statistical analysis.

For the primary efficacy endpoint analysis, the number and percentage of patients in each response category (i.e., responder, nonresponder, or indeterminate) for both treatment arms and also an unstratified 95% confidence interval (CI), according to Miettinen and Nurminen (42), were calculated. If the lower limit of the 95% CI for the treatment difference between rates of responders was greater than –10%, noninferiority of tedizolid to linezolid was concluded in the ITT population.

For the secondary efficacy endpoint analyses, the number and percentage of patients with programmatic or investigator-assessed clinical outcomes (i.e., clinical success, clinical failure, and indeterminate) for both treatment arms at the EOT and PTE visits were determined in the ITT, CE-EOT, and CE-PTE populations. Two-sided 95% CIs were computed for the observed treatment differences in the clinical success rates using the method of Miettinen and Nurminen (42), and noninferiority was concluded or rejected.

*Post hoc* analyses were performed in the mITT population, after exclusion of patients who did not receive any study drug, for early clinical response rate and investigator assessment of changes in local, regional, and systemic signs and symptoms of the primary ABSSSI lesion.

The safety parameters were evaluated by descriptive statistical methods.

All variables were analyzed by descriptive statistical methods. Missing data were not imputed for descriptive analyses. The number of data available, mean, standard deviation (SD), minimum, median, and maximum were calculated for continuous data. Frequency tables were generated for categorical data, and only patients with available data were included in the denominators. Statistical evaluation was performed using SAS, release 9.2 or higher (SAS Institute, Inc., Cary, NC, USA).

## Supplementary Material

Supplemental file 1
