# Supplementary material for: Efficacy and Safety of Tedizolid Phosphate versus Linezolid in a Randomized Phase 3 Trial in Patients with Acute Bacterial Skin and Skin Structure Infection
Source: Antimicrob Agents Chemother. 2019 Jun 24;63(7):e02252-18. doi: 10.1128/AAC.02252-18 (PMC6591607; doi:10.1128/AAC.02252-18)
Supplement: Supplemental file 1 [file AAC.02252-18-s0001.pdf]

Supplementary material

Full list of exclusion criteria

Patients who meet any of the following criteria are not eligible to participate in this study:

1. Uncomplicated skin and skin structure infections such as furuncles, minor abscesses (area of suppuration not surrounded by cellulitis/erysipelas), impetiginous lesions, superficial or limited cellulitis/erysipelas, and minor wound infections (e.g. stitch abscesses)
2. Infections associated with, or in close proximity to, a prosthetic device
3. Severe sepsis or septic shock
4. Known bacteremia at time of screening
5. Acute bacterial skin and skin structure infection (ABSSSI) due to or associated with any of the following:
   - Suspected or documented Gram-negative pathogens in patients with cellulitis/erysipelas or major cutaneous abscess that require an antibiotic with specific Gram-negative coverage. Patients with wound infections where Gram-negative adjunctive therapy is warranted may be enrolled if they meet the other eligibility criteria
   - Diabetic foot infections, gangrene, or perianal abscess
   - Concomitant infection at another site not including a secondary ABSSSI lesion (e.g. septic arthritis, endocarditis, osteomyelitis)
   - Infected burns
   - Decubitus or chronic skin ulcer, or ischemic ulcer due to peripheral vascular disease (arterial or venous)
   - Any evolving necrotizing process (i.e. necrotizing fasciitis)
   - Infected human or animal bites. However, arthropod (e.g. insects, spiders, 'bugs') bites are allowed only if subject actually witnessed the arthropod bite through the skin in the area of the ABSSSI; these are not considered animal bites in this study
   - Infections at vascular catheter sites or involving thrombophlebitis
   - Incision surgical site infection with any of the following characteristics:
     - Follows clean-contaminated surgery (urgent or emergency case that is otherwise clean, elective opening of respiratory, gastrointestinal, biliary, or genitourinary tract with minimal spillage [e.g. appendectomy] not encountering infected urine or bile; minor technique break)
     - Follows contaminated surgery (non-purulent inflammation; gross spillage from gastrointestinal tract; entry into biliary or genitourinary tract in the presence of infected bile or urine; major break in technique; chronic open wounds to be grafted or covered)

  - Follows dirty surgery (purulent inflammation [e.g. abscess]; preoperative perforation of respiratory, gastrointestinal, biliary, or genitourinary tract)
  - Extends into the fascia or muscle layers, organs, or spaces

6. Use of antibiotics as follows:
   - Systemic antibiotic with activity against Gram-positive cocci for the treatment of any infection within 24 hours before the first infusion of study drug
   - Patients who failed prior therapy for the primary infection site are also excluded from enrollment
   - Topical antibiotic on the primary lesion within 24 hours before the first infusion of study drug except for antibiotic/antiseptic-coated dressing applied to the clean post-surgical wound
7. Administration of linezolid within 30 days before the first infusion of the study drug
8. Recent history of opportunistic infections where the underlying cause of these infections is still active (e.g. leukemia, transplant, acquired immunodeficiency syndrome [AIDS])
9. Receiving chronic systemic immunosuppressive therapy such as prednisone doses ≥20 mg per day for ≥3 of the last 12 months or therapies that in the Investigator's judgement could predispose to opportunistic infections
10. Chronic (daily for the previous 30 days) use of antipyretic medication (e.g. acetaminophen, paracetamol, non-steroidal anti-inflammatory drugs). Low-dose aspirin (≤200 mg per day) for cardiovascular prophylaxis is allowed
11. Receiving treatment for active tuberculosis
12. Last known CD4 count <200 cells/mm^3 in patients with AIDS
13. Current or anticipated neutropenia with absolute neutrophil count <1000 cells/mm^3
14. Severe renal disease defined as creatinine clearance <30 mL/min estimated by the Cockcroft–Gault formula or requirement for peritoneal dialysis, plasmapheresis, hemodialysis, venovenous dialysis, or other forms of renal filtration
15. Alanine aminotransferase or aspartate aminotransferase ≥5 upper limit of normal or moderate-to-severe hepatic disease with Child–Pugh score ≥7 defined by the following:
   - Presence of ascites upon examination
   - Evidence of encephalopathy upon examination
   - Total bilirubin ≥2 mg/dL
   - Serum albumin ≤3.5 g/dL
   - Prothrombin time ≥4 seconds longer than control, or international normalized ratio ≥1.7
16. Significant or life-threatening condition or organ or system condition or disease (e.g. endocarditis, meningitis, unstable CNS conditions, acidosis or history of

lactic acidosis) that would confound or interfere with the assessment of the ABSSSI

17. Electrocardiogram finding of corrected QT interval >500 msec using either Bazett's correction method (QTcB) or Fridericia's correction method (QTcF)
18. In patients with uncontrolled hypertension, pheochromocytoma, carcinoid syndrome, or thyrotoxicosis, the use of the following medications within 2 days before the first infusion of study drug or planned use through the end of therapy (EOT) visit:
    - Systemic use of directly and indirectly acting sympathomimetic agents (e.g. pseudoephedrine, phenylpropanolamine), vasopressive agents (e.g. epinephrine, norepinephrine), or dopaminergic agents (e.g. dopamine, dobutamine). Use of a small amount of a vasoconstrictor (e.g. lidocaine containing epinephrine) during a minor surgical procedure under local anesthesia (e.g. incision and drainage) is allowed
19. Use of the following medications within 14 days before the first infusion of study drug or planned use through the EOT visit:
    - Monoamine oxidase A and B inhibitors (e.g. phenelzine, isocarboxazid)
    - Serotonergic agents including antidepressants such as selective serotonin reuptake inhibitors, tricyclic antidepressants, and serotonin 5-hydroxytryptamine receptor agonists (triptans), meperidine, or buspirone
20. High tyramine diet
21. Treatment with any investigational medicinal product within 30 days before the first infusion of study drug and previous assignment to treatment during this study
22. Investigational device present, or removed <30 days before the first infusion of study drug or presence of device-related infection
23. Previous inclusion in the tedizolid phosphate development programme
24. Hypersensitivity to oxazolidinones or any component in the formulation
25. If aztreonam adjunctive therapy is required in patients with wound infections, history of hypersensitivity to ceftazidime or any component of the aztreonam formulation
26. For patients with wound infections, history of hypersensitivity to metronidazole or any component of the formulation, if metronidazole adjunctive therapy is required
27. Patients who the Investigator considers unlikely to adhere to the protocol, comply with study drug administration, or complete the clinical study
28. Close affiliation with the investigational site (e.g. a close relative of the Investigator, dependent person [e.g. employee or student of the investigational site])

Detailed definitions of all secondary endpoints

Investigator's assessment of clinical response at 48–72-hour visit:

- Improving: improvement in the overall clinical status of the ABSSSI that was compatible with continuation of study drug therapy
- Stable: signs and symptoms were stable, no apparent change in the overall clinical status of the ABSSSI that was compatible with continuation of study drug therapy
- Failure: patient did not meet the requirements of 'Improving' or 'Stable' clinical response

Investigator's assessment of clinical response at Day 7 visit:

- Improving: improvement in the overall clinical status of the ABSSSI that was compatible with continuation of study drug therapy; a decrease in primary ABSSSI lesion size was assessed (area, length and width) compared with baseline; investigator assessment of tenderness was mild or absent; and no purulent drainage from a wound infection, or the purulent drainage was assessed with a lesser intensity compared with baseline/screening
- Failure: patient did not meet the requirements of 'Improving' clinical response

Programmatic objective clinical response at EOT (Day 11) visit:

- **Sustained clinical success** was defined if a patient was afebrile (<37.7°C oral, investigator reported) or fever ≥37.7°C was attributable to a cause other than the primary ABSSSI; a decrease in primary ABSSSI lesion size was assessed (area, length and width) compared with baseline; the investigator assessment of tenderness was mild or absent; and no purulent drainage from a wound infection or the purulent drainage was assessed with a lesser intensity compared with baseline/screening.
    - Additionally, the patient did not receive any systemic concomitant antibiotic treatment with activity against the baseline pathogen (with the exception of aztreonam and/or metronidazole for patients with wound infection); did not have a treatment-emergent adverse event leading to discontinuation of study drug requiring additional antibiotic therapy to treat their ABSSSI; did not receive any additional antibiotic to treat the primary ABSSSI; no unplanned major surgical intervention was performed to the primary ABSSSI; did not develop osteomyelitis after baseline; for a patient with a wound infection or an abscess, no incision plus drainage was performed after Day 1 unless it was planned at randomization; for a patient with cellulitis/erysipelas, no incision plus drainage was performed after the 48–72-hour visit.

- **Clinical failure** was defined if a patient was febrile (≥37.7°C oral, investigator reported) and fever was attributable to the primary ABSSSI; no decrease in primary ABSSSI lesion size compared with baseline was assessed; investigator assessment of tenderness was moderate or severe, or persistent purulent drainage from a wound infection at equivalent or greater intensity compared with baseline/screening.
  - Moreover, the patient received systemic concomitant antibiotic treatment with activity against the baseline pathogen (with the exception of aztreonam and/or metronidazole for patients with wound infection); or had a treatment-emergent adverse event leading to discontinuation of study drug requiring additional antibiotic therapy to treat their ABSSSI; or received any additional antibiotic to treat the primary ABSSSI; or an unplanned major surgical intervention was performed to the primary ABSSSI; or developed osteomyelitis after baseline; or for a patient with a wound infection or an abscess, incision plus drainage was performed after Day 1; or for a patient with cellulitis/erysipelas, an incision plus drainage was performed after the 48–72-hour visit; or death occurred within 28 days of the first infusion of study drug.
- **Indeterminate response** was defined if the patient had osteomyelitis at baseline; or was lost to follow-up or withdrew consent prior to EOT; or a Gram-negative pathogen was confirmed at baseline that required a different antibiotic therapy for patients with cellulitis or abscess or a different antibiotic therapy other than aztreonam and/or metronidazole for patients with wound infection.

Investigator's assessment of clinical response at EOT and post-therapy evaluation (PTE) visits:

- Patients were assessed as **clinical success** if they met all of the following criteria:
  - Resolution or near resolution of the most disease-specific signs and symptoms
  - Absence or near absence of regional and/or systemic signs of infection, if present at baseline
  - No new signs, symptoms, or complications attributable to primary ABSSSI lesion was present; thus, no further antibiotic therapy was required for the treatment of primary ABSSSI lesion
- Patients were assessed as **clinical failure** if they met any of the following criteria:
  - Required additional antibiotic therapy for the treatment of primary ABSSSI lesion
  - Unplanned major surgical intervention was performed due to failure of the study drug

    - Developed osteomyelitis after baseline
    - Treatment-emergent adverse event occurred leading to discontinuation of study drug and the patient required additional antibiotic therapy to treat the primary ABSSSI
    - Persistent Gram-positive bacteremia
    - Death occurred within 28 days of the first infusion of study drug
- Patients were assessed as **indeterminate** if they met any of the following criteria:
    - Osteomyelitis was present at baseline
    - Lost to follow-up
    - Withdrew consent
    - Extenuating circumstances that precluded the classification of a clinical success or clinical failure
    - A Gram-negative pathogen was confirmed at baseline that required a different antibiotic therapy for patients with cellulitis or abscess
    - A Gram-negative pathogen was confirmed at baseline that required a different antibiotic therapy other than aztreonam and/or metronidazole for patients with wound infection

Investigator's assessment of clinical response at late follow-up visit:

- Patients were assessed as sustained **clinical success** if no new signs or symptoms of the primary ABSSSI were present after the PTE visit
- Patients were assessed as **clinical failure** or relapse if new or worsened signs or symptoms of the primary ABSSSI were present after the PTE visit
- **Indeterminate** response was defined as study data were not available for the evaluation of efficacy outcome for any of the following reasons:
    - Lost to follow-up
    - Extenuating circumstances that precluded the classification of a clinical success or clinical failure
    - Withdrew consent

Table S1. List of reasons for screening failures

	**Reason**	**Number of patients**
1	ABSSSI due to or associated with any of the criteria as outlined among Exclusion criteria	4
2	Use of prohibited medications or antibiotics with activity against Gram-positive pathogens as outlined among Exclusion criteria	4
3	Presence of neutropenia as outlined in Exclusion criteria	1
4	Elevated liver enzymes as outlined in Exclusion criteria	7
5	Severe renal disease	1
6	Severe sepsis or septic shock	1
7	Significant life-threatening disease	2
8	Lack of compliance or adherence to protocol according to the Investigator	4
9	ABSSSI (i.e. cellulitis, erysipelas, wound infection, major cutaneous abscess) diagnosis could not be established	15
10	Lack of suspected or documented Gram-positive infection from baseline Gram stain or culture	2
11	Adequate venous access for a minimum of two IV doses of study drug	5
12	Unable to give written informed consent	1
13	Pregnancy or breastfeeding	1
14	Technical problems*	2
15	Withdrawal of consent for any reason based on GCP	7

ABSSSI: acute bacterial skin and skin structure infection; GCP: good clinical practice; IV: intravenous; IVRS: interactive voice response system

* Problems with IVRS and/or drug supply availability

Table S2. Lesion size parameters in patients with confirmed or suspected pathogens in different post-hoc analysis populations

	**Tedizolid phosphate**	**Linezolid**
**ITT population (all randomized patients)**		
**Overall**	**N=300**	**N=298**
Median	302.5	306.75
Mean (SD)	491.6 (618.1)	428.3 (391.7)
Range	75.0–6272.0	77.0–2664.0
**Patients with confirmed Gram-positive pathogen**	**N=113**	**N=126**
Median	288.0	263.25
Mean (SD)	532.5 (808.8)	405.7 (378.4)
Range	80.0–6272.0	77.0–2664.0
**Patients with suspected Gram-positive pathogen**	**N=187**	**N=172**
Median	308.0	331.6
Mean (SD)	466.9 (467.6)	444.8 (401.4)
Range	75.0–2745.6	77.0–2409.0
**modified ITT population (randomized patients excluding those who never received study drug)**		
**Overall**	**N=292**	**N=297**
Median	302.5	306.75
Mean (SD)	498.2 (624.7)	426.5 (391.1)
Range	75.0–6272.0	77.0–2664.0
**Patients with confirmed Gram-positive pathogen**	**N=110**	**N=125**
Median	293.5	262.5
Mean (SD)	542.7 (817.4)	401.3 (376.7)
Range	80.0–6272.0	77.0–2664.0
**Patients with suspected Gram-positive pathogen**	**N=182**	**N=172**
Median	308.0	331.6
Mean (SD)	471.2 (472.4)	444.8 (401.4)
Range	75.0–2745.6	77.0–2409.0

ITT: intent to treat; SD: standard deviation

Table S3. Investigator assessment of clinical response in the modified intent-to-treat (mITT) populations

**Response**	**Tedizolid phosphate N=292 n (%)**	**Linezolid N=297 n (%)**
**48–72 hours**		
Improvement in overall clinical status of ABSSSI compatible with continuation of study drug therapy	260 (89.0)	269 (90.6)
Signs and symptoms stable	14 (4.8)	9 (3.0)
Other[a]	2 (0.7)	0
**Day 7**		
Improvement in overall clinical status of ABSSSI compatible with continuation of study drug therapy	265 (90.8)	261 (87.9)
Other[b]	1 (0.3)	1 (0.3)

ABSSSI: acute bacterial skin and skin structure infection

[a] Signs and symptoms worsened but treatment was continued by investigator
[b] Signs and symptoms did not improve as assessed by investigator

Table S4. Improvements in regional/local signs in modified intent-to-treat population in post-hoc analyses

Parameters	Treatment	Day 2	48–72 h	Day 7	EOT	PTE
Lymphadenopathy absent, % (n/N1)	Tedizolid	37.1 (105/283)[a]	63.4 (175/276)	87.6 (233/266)	95.0 (265/279)	97.8 (262/268)
	Linezolid	37.3 (109/292)[b]	66.5 (183/275)	92.0 (241/262)	97.5 (274/281)	98.9 (261/264)
Lymph node tenderness absent, % (n/N1)	Tedizolid	38.2 (108/283)[c]	68.8 (190/276)	92.1 (245/266)	96.1 (268/279)	97.8 (262/268)
	Linezolid	39.2 (114/291)[d]	67.5 (185/274)	93.9 (246/262)	98.9 (278/281)	99.2 (262/264)
Erythema improved, % (n/N1)	Tedizolid	36.0 (102/283)[e]	77.5 (214/276)	94.0 (250/266)	92.5 (258/279)	96.7 (260/269)
	Linezolid	37.1 (109/294)[f]	75.4 (208/276)	95.8 (251/262)	95.0 (267/281)	98.5 (260/264)
Edema improved, % (n/N1)	Tedizolid	35.0 (99/283)[g]	73.9 (204/276)	94.0 (250/266)	93.9 (262/279)	96.7 (260/269)
	Linezolid	37.0 (108/292)[h]	78.5 (215/274)	95.4 (248/260)	95.0 (265/279)	98.5 (257/261)
Induration absent, % (n/N1)	Tedizolid	4.2 (12/283)[i]	6.5 (18/276)	18.8 (50/266)	29.0 (81/279)	42.2 (113/268)
	Linezolid	4.4 (13/293)[j]	8.3 (23/276)	22.2 (58/261)	33.2 (93/280)	45.6 (120/263)

EOT: end of therapy; PTE: post-therapy evaluation; n: number of observation; N1: number of patients with valid assessment at each time point

Number of patients with regional/local signs present, or mild/moderate/severe at baseline, respectively: [a]n=205; [b]n=209; [c]n=205; [d]n=208; [e]n=282; [f]n=293; [g]n=282; [h]n=289; [i]n=167; [j]n=173

Table S5. Lesion size changes in modified intent-to-treat population overall and with confirmed pathogens at baseline in post-hoc analyses

Mean change in lesion size, cm^2	Treatment	Baseline, mean (SD)	Day 2	48–72 h	Day 7	EOT	PTE
modified ITT population	Tedizolid	498.2 (624.7)	–79.2	–214.4	–342.6	–406.3	–457.0
	Linezolid	426.5 (391.1)	–83.2	–208.8	–332.3	–370.2	–407.2
modified Microbiological ITT population	Tedizolid	542.7 (817.4)	–77.3	–261.0	–374.6	–441.1	–497.8
	Linezolid	401.3 (376.7)	–75.4	–209.9	–324.7	–349.2	–387.9

EOT: end of therapy; ITT: intent to treat; PTE: post-therapy evaluation; SD: standard deviation

Table S6. Pain scores by visit [a] based on visual analogue scale score and Wong–Baker Faces Rating Scale score (modified intent-to-treat population)

**Visit**	**Tedizolid phosphate N=292**		**Linezolid N=297**	
	**Value at visit Mean (SD)**	**Change from baseline Mean (SD)**	**Value at visit Mean (SD)**	**Change from baseline Mean (SD)**
**VAS**				
Baseline	53.3 (27.2)		53.8 (28.7)	
Day 2	40.7 (26.2)	–12.8 (21.8)	39.9 (26.3)	–13.8 (20.1)
48–72 hours	29.7 (25.3)	–23.8 (23.9)	30.4 (25.4)	–23.3 (24.3)
Day 7	18.0 (22.2)	–35.5 (26.5)	17.5 (20.7)	–36.2 (26.6)
End of therapy	10.7 (18.1)	–42.6 (27.9)	10.0 (17.5)	–43.7 (29.1)
Post-therapy evaluation	10.7 (18.1)	–42.6 (27.9)	10.0 (17.5)	–43.7 (29.1)
**FRS**				
Baseline	5.6 (2.6)		5.7 (2.7)	
Day 2	4.1 (2.4)	–1.5 (2.1)	4.2 (2.5)	–1.5 (1.8)
48–72 hours	3.1 (2.3)	–2.5 (2.2)	3.2 (2.4)	–2.4 (2.3)
Day 7	2.0 (2.2)	–3.6 (2.5)	2.0 (2.1)	–3.6 (2.5)
End of therapy	1.2 (1.9)	–4.3 (2.7)	1.2 (1.8)	–4.4 (2.7)
Post-therapy evaluation	1.2 (1.9)	–4.3 (2.7)	1.2 (1.8)	–4.4 (2.7)

FRS: Wong–Baker faces rating scale; VAS: visual analogue scale

[a] Last observation carried forward

Table S7. Post-baseline substantially abnormal[a] clinical laboratory values (safety population)

**Preferred term**	**Tedizolid phosphate 200 mg, QD, 6 days N=292**	**Linezolid 600 mg, BD, 10 days N=297**
Alanine aminotransferase, n/N1 (%)	14/276 (5.1)	19/265 (7.2)
Aspartate aminotransferase, n/N1 (%)	6/271 (2.2)	12/261 (4.6)
Alkaline phosphatase, n/N1 (%)	2/278 (0.7)	0/269 (0)
Blood urea nitrogen, n/N1 (%)	0/278 (0)	0/270 (0)
Creatinine, n/N1 (%)	0/278 (0)	0/269 (0)
Hemoglobin, n/N1 (%)	0/270 (0)	2/263 (0.8)
Neutrophils, n/N1 (%)	0/270 (0)	3/262 (1.1)
Platelets, n/N1 (%)	2/257 (0.8)	1/252 (0.4)

N: total number of patients in safety population; N1: number of patients in the safety population with the pre-specified clinical laboratory value at baseline and post-baseline; BD: twice daily; QD: once daily

[a] Chemistry: alanine aminotransferase, aspartate aminotransferase, alkaline phosphatase, blood urea nitrogen, creatinine: substantially abnormal represents >2× upper limit of normal (ULN) for normal values at baseline; or >2× ULN and >2× baseline value for abnormal values at baseline. Hematology: substantially abnormal represents <75% of the lower limit of normal (LLN) for normal values of hemoglobin and platelet at baseline, or <75% of the LLN and <75% of baseline for abnormal values at baseline; or <50% of LLN for normal values of absolute neutrophil count at baseline, or <50% of the LLN and <50% of baseline for abnormal values at baseline.

Table S8. List of study investigators

	Surname, Given name	Hospital	City, Country
1	Lv, Xiaoju	West China School of Medication, West China Hospital, Sichuan University	Chengdu, China
2	Bao, Wanguo	The First Hospital of Jilin University	Changchun, China
3	Chen, Jianghan	Shanghai Changzheng Hospital	Shanghai, China
4	Chen, Qilong	The First Teaching Hospital of Xinjiang Medical University	Urumqi, China
5	Cheng, Hao	Sir Run Run Shaw Hospital, Medical School of Zhejiang University	Hangzhou, China
6	Fang, Ruihua	Guangzhou First People's Hospital	Guangzhou, China
7	Feng, Wenli	The 2nd Hospital of Shanxi Medical University	Taiyuan, China
8	Guo, Zaipei	West China School of Medication, West China Hospital, Sichuan University	Chengdu, China
9	He, Li	The 1st Affiliated Hospital of Kunming Medical University	Kunming, China
10	Hu, Zhiqiang	The Third Hospital of Changsha	Changsha, China
11	Huang, Feizhou	The Third Xiangya Hospital of Central South University	Changsha, China
12	Huang, Jinhua	The Third Xiangya Hospital of Central South University	Changsha, China
13	Huang, Zhongcheng	Hunan Provincial People's Hospital	Changsha, China
14	Ji, Bihua	Yijishan Hospital Affiliated to Wannan Medical College	Wuhu, China
15	Li, Jun	Jiangsu Province Hospital	Nanjing, China
16	Li, Shenqiu	Tongji Hospital, Tongji Medical College of Huazhong University of Science & Technology	Wuhan, China
17	Liu, Quanzhong	Tianjin Medical University General Hospital	Tianjin, China
18	Lu, Jianguo	Tangdu Hospital	Xi'an City, China
19	Mou, Kuanhou	The 1st Affiliated Hospital of Xi'an Jiaotong University	Xi'an City, China
20	Paride, Abliz	The First Teaching Hospital of Xinjiang Medical University	Urumqi, China
21	Pan, Weili	Zhejiang Provincial People's Hospital	Hangzhou, China
22	Qian, Qihong	The First Affiliated Hospital of Suzhou	Suzhou, China

		University	
23	Shan, Yuanzhou	Shanghai Fengxian District Central Hospital	Shanghai, China
24	Shen, Weixing	Zhongshan Hospital Fudan University, Qingpu Branch	Shanghai, China
25	Sun, Qing	Qilu Hospital, Shandong University	Jinan, China
26	Sun, Qiuning	Peking Union Medical College Hospital	Beijing, China
27	Wang, Lie	Fuzhou General Hospital of Nanjing Military Command	Fuzhou, China
28	Wang, Liming	The Second Hospital of Dalian Medical University	Dalian, China
29	Wei, Hongxia	The Second Hospital of Nanjing	Nanjing, China
30	Xia, Jiazeng	Wuxi No.2 People's Hospital	Wuxi, China
31	Yang, Xiumin	Beijing Tongren Hospital Capital Medical University	Beijing, China
32	Yao, Zhirong	Xinhua Hospital Affiliated to Shanghai Jiaotong University School of Medicine	Shanghai, China
33	Ye, Hui	West China School of Medication, West China Hospital, Sichuan University	Chengdu, China
34	Zha, Xushan	The 1st Affiliated Hospital of Guangzhou Chinese Medicine University	Guangzhou, China
35	Zhang, Chunlei	Peking University Third Hospital	Beijing, China
36	Zhang, Jie	Putuo District Central Hospital	Shanghai, China
37	Zhang, Shifa	The General Hospital Of Shenyang Military Region	Shenyang, China
38	Zhao, Junying	Beijing Friendship Hospital, Capital Medical University	Beijing, China
39	Zhao, Yongjie	Tianjin Union Medical Center	Tianjin, China
40	Zheng, Jie	Ruijin Hospital, Shanghai Jiao Tong University School of Medicine	Shanghai, China
41	Zheng, Min	The Second Hospital Affiliated to Zhejiang University School of Medicine	Hangzhou, China
42	Chang, Shan-Chwen	National Taiwan University Hospital	Taipei, Taiwan
43	Chen, Yao-Shen	Veterans General Hospital-Kaohsiung	Kaohsiung, Taiwan
44	Chen, Yen-Hsu	Kaohsiung Medical University Chung-Ho Memorial Hospital	Kaohsiung, Taiwan
45	Lee, Wen-Sen	Taipei Municipal Wanfang Hospital	Taipei, Taiwan

46	Tang, Hung-Jen	Chi- Mei Medical Hospital, Tai-Nan Hsien	Tainan, Taiwan
47	Wang, Jen-Hsien	China Medical University Hospital	Taichung, Taiwan
48	Esguerra, Enrique	St. Luke's Medical Center	Taguig City, Philippines
49	Rosario, Minette	University of East Ramon	Quezon City, Philippines
50	Birch, Thomas	Holy Name Medical Center	Teaneck, NJ, USA
51	Green, Sinikka	eStudySite	La Mesa, CA, USA
52	Kabler, Heidi	eStudySite	Las Vegas, NV, USA
53	Mannis, Steven	Tri-City Medical Center	Oceanside, CA, USA
54	Manos, Paul	Tri-City Medical Center	Oceanside, CA, USA
55	Overcash, J Scott	eStudySite	Chula Vista, CA, USA
